# Mental health and self-management in glaucoma patients during the COVID-19 pandemic: a cross-sectional study in China

**DOI:** 10.1186/s12886-022-02695-2

**Published:** 2022-12-06

**Authors:** Wenzhe Zhou, Haishuang Lin, Yanhan Ren, Hao Lin, Youping Liang, Yanyan Chen, Shaodan Zhang

**Affiliations:** 1 The Eye Hospital, School of Ophthalmology and Optometry, Wenzhou Medical University, Wenzhou, 325027 Zhejiang Province China; 2 Wenzhou Medical University, Wenzhou, 325027 Zhejiang Province China; 3 University of Massachusetts Medical School, Worcester, USA

**Keywords:** Anxiety, Depression, Coronavirus Disease 2019, Glaucoma, Self-Management

## Abstract

**Purpose:**

To investigate mental health and self-management in glaucoma patients during the COVID-19 pandemic in China and to describe the correlation between anxiety, depression, glaucoma, and self-management.

**Methods:**

This cross-sectional study included glaucoma patients who enrolled in the case management platform and completed an online survey. The survey included the Generalized Anxiety Disorder (GAD-7), Patient Health Questionnaire (PHQ-9), and Glaucoma Self-Management Questionnaire (GSMQ).

**Results:**

Among 109 glaucoma patients enrolled in this study, the proportions of patients suffering from depression and anxiety during the COVID-19 pandemic were 26.6% and 20.2%, respectively. A statistical association was found between depression and self-management behaviour in these glaucoma patients (*r *= -0.247, *P* = 0.010). The self-management scores in patients less than 35 years were lower than those in patients aged 35–60 years (*P* = 0.046). The scores of body function promotion in men were lower than those in women (*P* = 0.048). Patients with primary school education and below had lower scores in the medical management of disease than those with either middle school education (*P* = 0.032) or community college education or higher (*P* = 0.022).

**Conclusion:**

A high proportion of anxiety and depression was found in glaucoma patients during the COVID-19 pandemic. Better self-management behaviour was associated with stronger mental health regulation. It is important to help glaucoma patients improve their self-management behaviours, especially for young men with low educational levels.

## Introduction

Glaucoma is a chronic ocular disorder characterized by progressive optic nerve damage leading to irreversible visual field defects [1]. In 2020, it was estimated that 80 million people had glaucoma worldwide, but this number is expected to increase to over 11.18 million by 2040 [2]. Glaucoma has considerable economic and psychological burdens on patients [3, 4]. It has been reported that there is a statistically significant association between glaucoma and anxiety and depression [5]. Since the therapeutic approach to glaucoma involves control of intraocular pressure (IOP) through the use of a combination of drugs, underlying anxiety and depression in glaucoma patients may cause poor adherence to treatment and thus hasten disease progression [6]. A health psychology approach to glaucoma therapy should pay attention to the management of mental health issues associated with the disease [6]. China has faced the major challenge of the coronavirus disease 2019 (COVID-19) pandemic since December 2019 [7]. The Chinese government has imposed strict restrictions to control the epidemic, such as social isolation, quarantine and city-wide lockdown, since January 2020. The lockdown disrupted follow-up plans for glaucoma patients, leaving most patients unable to access in-person care. The ability to buy eyedrops and monitor intraocular pressure were limited, which may affect the mental health of glaucoma patients through anxiety and depression.

Self-management support is a behavioural strategy that maintains healthy outcomes and enhances one’s condition by monitoring and regulating behaviours and reinforcements. Recent research has suggested that self-management support intervention has a favourable impact on improving quality of life and can reduce the incidence of depression and anxiety among patients with chronic disorders such as diabetes, stroke, nephropathy, and age-related macular degeneration [8–13]. Self-management may play a positive role in the mental health of glaucoma patients. However, there are few reports about the psychological burdens and self-management of glaucoma patients. The scale for Generalized Anxiety Disorder (GAD-7) is a valid and efficient tool for screening for generalized anxiety disorder and assessing its severity in clinical practice and research, with excellent internal reliability (Cronbach’s α = 0.92) and good test–retest reliability (intraclass correlation = 0.83) [14]. The Patient Health Questionnaire (PHQ-9) is a validated, easily administered screening questionnaire for depression with excellent internal reliability (Cronbach’s α = 0.89) and good test–retest reliability (intraclass correlation = 0.84) [15]. As a recommended tool for evaluating self-management behaviour among glaucoma patients, the Glaucoma Self-management Questionnaire (GSMQ) has been found to exhibit good reliability and validity. The content validity index of the GSMQ was 0.98, the Cronbach’s α for the three factors ranged from 0.712 to 0.891, and the test–retest reliabilities ranged from 0.612 to 0.833 [16]. In this study, we investigated the anxiety, depression and self-management scores of glaucoma patients during the COVID-19 pandemic via the GAD-7, PHQ-9 and GSMQ and explored the association between self-management and mental health.

## Methods

### Study participants and survey method

This cross-sectional study recruited patients aged 18 years and older from the glaucoma clinics of the Eye Hospital of Wenzhou Medical University. Patients were included if they were managed with medical therapy and voluntarily joined the case management platform with regular follow-ups. Participants were excluded if they had any prior ocular surgery in the last 2 months or had cognitive disorder. We mainly focused on glaucoma patients with medication treatment and regular follow-up. Therefore, only patients with primary open angle glaucoma (78.4%) and those with chronic angle closure glaucoma (CACG, 21.5%) were included in the present study. Since CACG was less common, we did not perform further analysis according to different glaucoma subtypes. WeChat-based electronic questionnaires were sent to enrolled participants from March 13^th^, 2020, to March 16^th^, 2020. The purpose of this study was introduced to all participants with standard instructions by the assigned case manager, and informed consent was obtained prior to their participation. To assure the effectiveness and integrity of this study, all questions were compulsory, and each respondent, identified by phone number, could respond to the questionnaire only once. A total of 109 questionnaires were sent out, and 109 questionnaires were returned (response rate: 100%). The logic of the questionnaires was checked by a case manager before data analysis. This study was approved by the Ethics Committee of the Eye Hospital of Wenzhou Medical University (2020–031-K-29–01) and conducted according to the tenets of the Declaration of Helsinki.

### Assessment of mental health and self-management

The GAD-7 comprises 7 questions based on the Diagnostic and Statistical Manual of Mental Disorders, Fourth Edition (DSM-IV) symptom criteria for GAD. The scale of each question ranges from 0 (not at all) to + 3 (nearly every day). The total score was calculated by summing responses to the 7 items. Total scores of 0–4, 5–9, 10–14, and 15–21 were interpreted as representing minimal, mild, moderate, and severe levels of anxiety on the GAD-7, respectively.

The PHQ-9 is composed of 9 items based on DSM-IV symptom criteria for depression disorder, which scores each of the 9 DSM-IV criteria as 0 (not at all) to + 3 (nearly every day). The total score ranges from 1 to 27. Cut points of 5, 10, and 15 represent mild, moderate, and severe levels of depression, respectively.

The GSMQ includes 17 items assessing the following 3 domains: life adjustment, body function promotion, and medical management of disease. A 4-point Likert scale is used for each item. The scale range is 1 (totally cannot do it) to + 4 (totally can do it). The total score is calculated by summing responses to the 17 items. A higher total score indicates higher self-management.

### Statistical analysis

Statistical analysis was performed using SPSS Statistics (version 22.0, IBM, USA). Characteristics of the study population were examined using proportions, means, and standard deviations. The correlation between self-management and mental health was examined using Pearson correlation. Multiple linear regression analysis was used with self-management as the dependent variable and demographic characteristics such as gender and age as the independent variables. All *p* values (P) were 2-sided and considered statistically significant when less than 0.05.

## Results

A total of 109 glaucoma patients were enrolled in this study (62 men and 47 women; mean age 45 ± 16 years). The demographic data of the total sample are presented in Table 1.

**Table 1:** Demographic characteristics of participants

	**N**	**%**	
Gender			
Men	62	56.9	
Women	47	43.1	
Education level			
Primary school and below	20	18.3	
Middle school	46	42.2	
Community college or higher	43	39.4	
Disease course duration			
≤ 1 year	29	26.6	
2–4 years	37	33.9	
≥ 5 years	43	39.4	
Work or not			
Yes	78	71.6	
No	31	28.4	
Habitant			
Wenzhou	76	69.7	
Outside Wenzhou	33	30.3	
Live alone			
Yes	8	7.3	
No	101	92.7	

### Anxiety, depression, and self-management

During the COVID-19 pandemic public emergency, 50 (45.9%) glaucoma patients self-reported suffering from a higher level of anxiety, whereas 29 (26.6%) patients suffered from a higher level of depression. The mean GAD-7 and PHQ-9 scores in the study population were 2.1 ± 3.3 and 3.1 ± 3.9, respectively. The proportions of depression and anxiety were 26.6% (n = 29) and 20.2% (n = 22), respectively. Most participants had a mild level of depression (22/29, 75.9%) and a minimal level of anxiety (18/22, 81.8%).

The mean GSMQ score in these 109 glaucoma patients was 54.1 ± 7.0. Among the three dimensions of the GSMQ, the score of medical management of disease was highest (27.4 ± 3.9) followed by body function promotion (17.8 ± 3.0), and life adjustment was lowest (8.9 ± 1.5).

Not surprisingly, the depression measure (PHQ-9) strongly correlated with the total GSMQ score (*r* = -0.247, *P* = 0.010) in patients suffering from glaucoma. In three dimensions of the GSMQ, a negative correlation between body function promotion and symptoms of depression was found (*r* = -0.236, *P* = 0.014), and a negative correlation with the life adjustment dimension was found for the severity of either anxiety (*r* = -0.314, *P* = 0.001) or depression (*r* = -0.344, P < 0.001). However, the total GSMQ scores did not have significant associations with anxiety (*r* = -0.159, *P* = 0.100) (Table 2).

**Table 2:** Correlation analysis of self-management and three dimensions with anxiety, depression in glaucoma patients

	**Life adjustment**		**Body function promotion**		**Medical management of disease**		**Self-management**	
	***r* value**	***P* value**	***r* value**	***P* value**	***r* value**	***P* value**	***r* value**	***P* value**
Anxiety	-0.314	0.001	-0.134	0.166	-0.056	0.563	-0.159	0.100
Depression	-0.344	< 0.001	-0.236	0.014	-0.124	0.199	-0.247	0.010

### Risk factors of self-management

Multiple linear regression analysis was used to analyse the association between self-management and demographic characteristics. The GSMQ scores in patients less than 35 years (52.6 ± 7.6) were lower than those in patients aged 35–60 years (55.1 ± 6.4) (t = -2.023, *P* = 0.046). Patients who self-reported feeling more depressed (50.6 ± 6.41) during the COVID-19 pandemic had lower GSMQ scores than those without more depressive feelings (55.4 ± 7.0) (t = -2.263, *P* = 0.026). The scores of body function promotion in men (17.3 ± 2.8) were significantly lower than those in women (18.5 ± 3.2) (t = 2.004, *P* = 0.048). The scores in patients aged < 35 years (16.3 ± 3.3) were lower than those aged 35–60 years (18.3 ± 2.6) (t = -2.971, *P* = 0.004). Patients with primary school education and below (25.6 ± 4.9) had lower scores than those with either middle school education (28.0 ± 3.2) (t = 2.175, *P* = 0.032) or community college education or higher (27.7 ± 4.0) (t = 2.321, *P* = 0.022) in the medical management of disease (Table 3).

**Table 3:** Multilinear regression analysis of risk factors in self-management in glaucoma patients

**Dependent variable**	**Independent variable**		**β value**	**SE**	**β'value**	**T value**	***P* value**
Self-management^a^	Age	35–60 years	ref				
		< 35 years	-3.525	1.742	-0.225	-2.023	0.046
		> 60 years	0.497	2.116	0.030	0.235	0.815
	Gender	Men	ref				
		Women	2.353	1.446	0.167	1.627	0.107
	Education level	Primary school and less	ref				
		Middle school	3.768	2.079	0.266	1.813	0.073
		Community college or higher	3.536	2.277	0.247	1.553	0.124
	Feel more anxious than before	No	ref				
		Yes	-1.512	1.659	-0.108	-0.911	0.365
	Feel more depressive than before	No	ref				
		Yes	-4.35	1.922	-0.275	-2.263	0.026
Life adjustment^b^	Age	35–60 years	ref				
		< 35 years	-0.307	0.405	-0.089	-0.759	0.45
		> 60 years	-0.313	0.491	-0.086	-0.636	0.526
	Gender	Men	ref				
		Women	-0.111	0.336	-0.036	-0.33	0.742
	Education level	Primary school and less	ref				
		Middle school	-0.037	0.483	-0.012	-0.076	0.939
		Community college or higher	-0.292	0.529	-0.093	-0.552	0.582
	Feel more anxious than before	No	ref				
		Yes	-0.259	0.385	-0.084	-0.672	0.503
	Feel more depressive than before	No	ref				
		Yes	-0.628	0.446	-0.18	-1.407	0.163
Body function promotion^c^	Age	35–60 years	ref				
		< 35 years	-2.226	0.749	-0.328	-2.971	0.004
		> 60 years	0.666	0.910	0.094	0.732	0.466
	Gender	Men	ref				
		Women	1.246	0.622	0.203	2.004	0.048
	Education level	Primary school and less	ref				
		Middle school	1.265	0.894	0.206	1.415	0.160
		Community college or higher	0.861	0.979	0.139	0.879	0.382
	Feel more anxious than before	No	ref				
		Yes	-0.858	0.714	-0.141	-1.202	0.232
	Feel more depressive than before	No	ref				
		Yes	-1.133	0.827	-0.165	-1.371	0.174
Medical management of disease^d^	Age	35–60 years	ref				
		< 35 years	-0.992	0.979	-0.113	-1.013	0.313
		> 60 years	0.144	1.189	0.016	0.121	0.904
	Gender	Men	ref				
		Women	1.217	0.812	0.154	1.499	0.137
	Education level	Primary school and less	ref				
		Middle school	2.540	1.168	0.321	2.175	0.032
		Community college or higher	2.967	1.279	0.371	2.321	0.022
	Feel more anxious than before	No	ref				
		Yes	-0.395	0.932	-0.05	-0.424	0.673
	Feel more depressive than before	No	ref				
		Yes	-2.588	1.080	-0.293	-2.398	0.018

^a^*R* = 0.450, R^2^ = 0.202, Adjusted R^2^ = 0.102, F = 2.027, *P* = 0.030

^*b*^*R* = 0.338 R^2^ = 0.114, Adjusted R^2^ = 0.004, F = 1.032 *P* = 0.427

^*c*^*R* = 0.465, R^2^ = 0.216, Adjusted R^2^ = 0.118, F = 2.202, *P* = 0.017

^*d*^*R* = 0.440, R^2^ = 0.194, Adjusted R^2^ = 0.093, F = 1.923, *P* = 0.041

## Discussion

The results of our study revealed that the psychological burden of anxiety and depression was particularly serious during the COVID-19 epidemic period. The proportion of generalized anxiety disorder among glaucoma patients in this study was 20.2%, which was higher than that in the United States (17.1%) [5], Turkey (14.0%) [17], Japan (13.0%) [18], and Europe (6.6%) [19] before the COVID-19 epidemic. The proportion of depression was 26.6%, which was at a high level compared with previous studies in the United States (22.0%) [5], Australia (19.1%) [20], China (16.40%) [21], Hungary (12.1%) [22], Japan (10.9%) [18], and countries in Europe (5.3%) [19] before the COVID-19 epidemic. Studies in Italy [23] and Turkey [24] also showed that people with chronic diseases had a higher proportion of anxiety and depression during the COVID-19 epidemic period. Cecchetto C and colleagues reported significantly decreased scores of depression (by GAD and PHD) among Italian residents after the restriction policy compared to those obtained during restriction (2.04 to 1.82 and 2.18 to 1.86, respectively) [25]. A study before COVID-19 on mental health in Chinese patients with glaucoma in Shanghai, a city near Zhejiang Province, reported a much lower prevalence of anxiety than the present study (12.11% vs. 20.2%) [26], which indirectly supports the influence of this pandemic on mental health in glaucoma patients. Although depression and anxiety were also increased among normal people during the pandemic, we propose that anxiety due to the inaccessibility of in-person care, eye drops and disease monitoring may greatly affect the self-management of patients and therefore the trend of disease progression. In addition, several reports have been addressed the anti-glaucoma medication on the mental health of glaucoma patients. Robin AL [27] reported that alpha-Agonists may cause psychologic depression and fatigue. Consistently, Enyedi LB et al [28] also suggested that brimonidine can passe through the blood–brain barrier, potentially causing central nervous system depression. Given the increasing proportion of anxiety and depression, more attention should be given to glaucoma patients both during and after pandemics.

The diagnosis, treatment and management of patients with chronic disease have changed considerably because of the COVID-19 epidemic. The promotion of a diabetes self-management educational programme for the whole country was proposed in an Indian study to achieve better control of blood glucose during the COVID-19 epidemic [29]. The combination of online and offline medical care for glaucoma patients was also recommended in China and Singapore [30]. Patients can research disease aetiologies or report disease outcomes by themselves based on new digital technologies such as smartphone applications and artificial intelligence programs. This behavioural strategy is called self-management. It has been reported that self-management plays a positive role in quality of life in many chronic diseases [31, 32]. Facilitating self-management behaviour can improve mental health in patients with chronic kidney disease and stroke [10, 13]. Consistent with previous research, our study found that self-management was negatively correlated with depression in glaucoma patients. From our viewpoint, the proportion of anxiety and depression increased because of COVID-19, whereas self-management was slightly influenced by COVID-19. The pandemic may have aggravated depression in patients with low levels of self-management. In view of the important role of self-management in the treatment of glaucoma patients, clinicians should pay attention to the self-management level of glaucoma patients when treating the disease. Questionnaires for self-management should be integrated into the workflow in glaucoma clinics. Thus, clinicians can provide targeted guidance in the weak dimensions according to the self-management level of patients, which may improve their ability to deal with the unknown during treatment. Furthermore, the assessment of mental health for patients with low levels of self-management may be of great use during treatment.

There was an interesting finding in this study. In the three dimensions of self-management, both life adjustment and bodily function promotion were negatively correlated with depression, whereas medical management showed no statistical association with depression. This may be because the case management platform in this study can provide the whole process of medical care planning and illness consultation with continuous monitoring and follow-up [3334]. Case managers can connect with patients closely through the platform. Knowledge of glaucoma was sent to patients who joined the case management platform to improve their medical management. Medical management was carried out well, with the highest scores in this study and higher scores than glaucoma patients in another study [31]. Consistent with the results of a previous study [31], the score of life adjustment in self-management was the lowest in our study. The possible reason is that Chinese clinicians usually pay more attention to disease knowledge and medical management but ignore the changes in life adjustment caused by glaucoma. As an important indicator of glaucoma, IOP can be affected by daily activities [35], such as resistance training [36, 37], frequent drinking [38], and head-down [39] or supine body positions [40]. Therefore, it is necessary to provide advice about life adjustment for glaucoma patients. Self-management scores were much higher in patients aged 35–60 years than in those < 35 years in this study, while body function promotion scores were higher in patients aged 35–60 years than in those < 35 years. Patients aged 35–60 years are the mainstay of the family and play an integral role in the family and society. These major roles encourage them to manage new chronic disease. On the other hand, they are more likely to have comorbidities and pay more attention to self-management than young patients. In contrast, young patients tend to stay up late, spend more time working and ignore their health due to work-related stress. Given this finding, a particular focus on the life adjustment of young patients is warranted to slow the progression of glaucoma to the greatest extent.

The results from our study also revealed gender differences in self-management. Higher self-management scores in women were consistent with a previous study [41]. In traditional Chinese values, women are more likely to take care of the family than men. Women tend to engage in positive health behaviour [42, 43], leading to variation in the composition of self-management behaviour by gender. On the other hand, patients with higher educational levels had higher self-management scores. Patients with higher educational levels are willing to take initiative to gain more knowledge about disease and management. Consistent with other researchers [43, 44], patients in our study with higher educational levels could better understand the importance of medical adherence and self-management of their medical conditions. Considering that patients' educational levels are associated with better medical management and slowed disease progression [45, 46], health care workers should pay attention to patients’ knowledge of glaucoma. For patients with low educational levels, new forms of education, such as simplified words and illustrations, are needed.

Limitations of the present study should be mentioned. First, anxiety and depression were measured by the GAD-7 and PHQ-9, respectively, rather than clinical assessments by mental health experts. However, the purpose of this study was to measure the symptomology of anxiety and depression, not to diagnose them. An ophthalmologist can screen for mental health difficulties and subsequently refer patients to psychiatrists. Second, this was a cross-sectional study. The glaucoma diagnosis, anti-glaucoma medications, and the optic nerve health situation were not collected, which may have different effects on mental status. In future work, we will investigate whether and how mental health status is related to self-management and disease progression.

## Conclusion

The proportion of anxiety and depression among glaucoma patients was higher than usual during the COVID-19 pandemic in China. Self-management was negatively correlated with depression in glaucoma patients. Questionnaires for self-management may be integrated into the workflow in glaucoma clinics. It is necessary to help glaucoma patients perform self-management behaviours via self-management education, especially for young men with low educational levels.

## Data Availability

The datasets used and/or analyzed during the current study available from the corresponding author upon reasonable request.
